# Hydrogen Behavior and Mechanism of Cr-Coated Zirconium Alloy Cladding During Simulated LOCA Tests

**DOI:** 10.3390/ma19153156

**Published:** 2026-07-23

**Authors:** Shen Li, Xin Liu, Yong Hu

**Affiliations:** China Institute of Atomic Energy, Beijing 102413, China; lishen@cnncmail.cn (S.L.); liuxin@cnncmail.cn (X.L.)

**Keywords:** Cr-coated zirconium alloy, hydrogen absorption, LOCA, microstructure

## Abstract

Extensive research has been conducted on accident-tolerant coatings for zirconium alloy claddings, while study of the hydrogen behavior during simulated loss-of-coolant accidents (LOCAs) has seemingly been overlooked. However, research on the effect of hydrogen on the performance of zirconium alloy claddings is crucial. Therefore, simulated LOCA tests with different oxidation durations were performed on uncoated and Cr-coated claddings. A series of microstructural analysis techniques were used to characterize the distribution of hydrogen and oxygen elements, as well as the evolution of microstructural characteristics, aiming to explore the hydrogen behavior and mechanism of Cr-coated zirconium alloy claddings during simulated LOCA tests. The results showed that no hydrogen absorption occurred in the uncoated claddings during the tests. The uncoated claddings formed a relatively simple microstructure with dense zirconia that prevented hydrogen diffusion into the zirconium alloy matrix. In contrast, severe hydrogen absorption occurred in the Cr-coated claddings. A complex microstructure consisting of interleaved oxide and metallic phases was formed in the Cr-coated claddings during the tests, which facilitated hydrogen absorption into the cladding matrix and is the main reason for the severe hydrogen absorption of Cr-coated claddings during LOCAs.

## 1. Introduction

Since the Fukushima nuclear accident in 2011, the accident tolerance of water reactor fuel claddings under LOCAs has become a research hotspot in the field of nuclear materials. To avoid similar accidents, “Accident-Tolerant Fuel (ATF)” claddings have been developed.

Traditional zirconium alloys are susceptible to loss-of-coolant accidents (LOCAs). During LOCAs, zirconium alloys undergo severe embrittlement and are prone to rupture, leading to serious nuclear leakage accidents. In severe cases, hydrogen produced by the zirconium–water reaction and a large amount of heat may even cause a severe reactor explosion. Depositing protective coatings on the surface of zirconium alloys can effectively reduce the oxidation rate, decrease hydrogen production, improve the residual ductility of materials after accidents, and provide a larger safety margin for accident conditions.

Therefore, in recent years, various countries have carried out extensive research on accident-tolerant coatings for zirconium alloy claddings [1]. Coatings such as Cr coatings [2,3,4], MAX phase coatings [5,6], and high-entropy alloy coatings [7,8,9,10] have received widespread attention and research. Among them, Cr coatings are considered the optimal choice for zirconium alloy claddings due to their excellent oxidation and corrosion resistance, good mechanical properties, radiation resistance, and thermal expansion coefficient compatible with that of zirconium alloys. However, most existing domestic and foreign studies on the protective effect of coatings have focused on oxidation resistance [11,12], water corrosion resistance [13], and fretting wear resistance [14,15]. Systematic investigations on the evolution of the hydrogen content in Cr-coated claddings remain relatively scarce. Although hydrogen embrittlement in conventional uncoated zirconium alloy claddings under loss-of-coolant accident (LOCA) conditions has been thoroughly demonstrated [16], Cr-coated claddings possess unique microstructural features such as grain boundaries and ZrCr_2_ intermetallic phases that act as diffusion and trapping pathways for hydrogen. Consequently, their hydrogen uptake characteristics differ markedly from those of zirconium alloys. Hence, specialized research on the hydrogen behavior of Cr-coated zirconium alloys under LOCA conditions is urgently required.

In this study, simulated LOCA tests with different oxidation degrees were conducted on uncoated and Cr-coated zirconium alloy claddings under the same oxidation conditions. The hydrogen content and distribution, oxygen content and distribution, and microstructure of the two types of claddings after simulated LOCA tests were characterized. The hydrogen behavior and mechanism of Cr-coated zirconium alloy claddings during simulated LOCA tests were analyzed.

## 2. Experimental Materials and Methods

### 2.1. Experimental Materials

**Figure 1:**
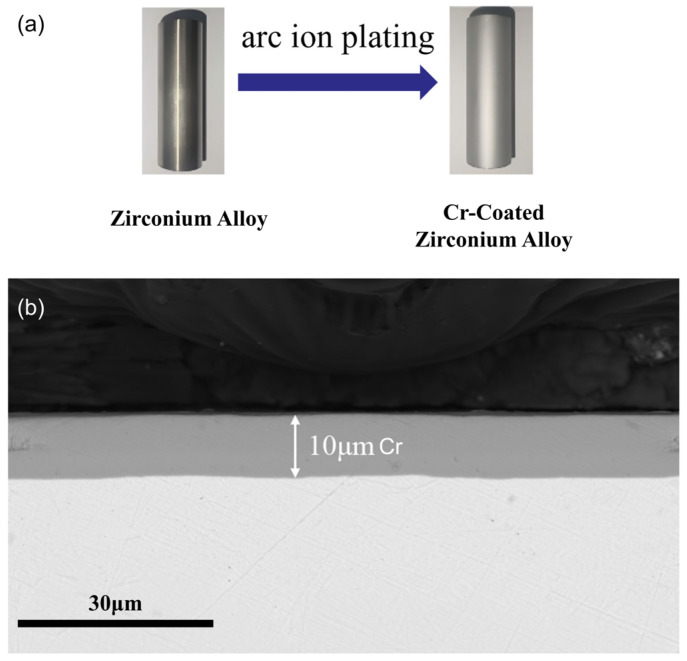
(**a**) Macroscopic morphologies of zirconium alloys and Cr-coated zirconium alloy; (**b**) cross-sectional morphology of Cr-coated zirconium alloy (SEM).

Zr-Sn-Nb alloy was used in this experiment, and its chemical composition is shown in Table 1. Cr-coated claddings were prepared by arc ion plating. The current of the chromium target was set to 100 A. The volume ratio of nitrogen to argon (N_2_:Ar) was 1:1, and the total gas pressure was maintained at 1.0 Pa. The substrate bias voltage was −50 V with a duty cycle of 70%, and the total deposition time lasted 70 min, resulting in a Cr coating with a thickness of 10 μm, as shown in Figure 1.

Uncoated and Cr-coated zirconium alloy cladding samples were cleaned with ethanol, dried, and weighed for subsequent tests.

### 2.2. High-Temperature Steam Oxidation and Quenching Tests

High-temperature steam oxidation tests were performed using a self-developed high-temperature steam oxidation test device. The test process can simulate the actual rapid heating condition of a LOCA to the maximum extent [17]. The oxidation temperature in this study was 1200 ± 3 °C; the heating rate was greater than 20 °C/s below 1000 °C and greater than 2 °C/s above 1000 °C. The steam flow rate during the test was 360 L/h. After oxidation, the temperature was cooled to 800 °C for quenching, and the time from 1200 °C to 800 °C was 100 s.

In the study of LOCA criteria for zirconium alloys, the concept of Equivalent-Cladding Reacted (ECR) is usually introduced [17], and the currently widely used CP-ECR [18] is calculated using the Cathcart–Pawel relationship. For the double-sided oxidation adopted in this experiment, the relationship between CP-ECR and the oxidation duration is

$$ECR=\frac{52.776}{n}exp(\frac{-10050}{T})\sqrt{t}$$

where *n* (cm) is the thickness of the cladding tube, *T* (K) is the oxidation temperature, and *t* (s) is the oxidation duration. The oxidation duration of the test was determined by this relationship, and the temperature change during the heating stage was processed by integration. The oxidation durations selected in this test were 112 s, 332 s, 730 s and 1020 s.

### 2.3. Ring Compression Test

**Figure 2:**
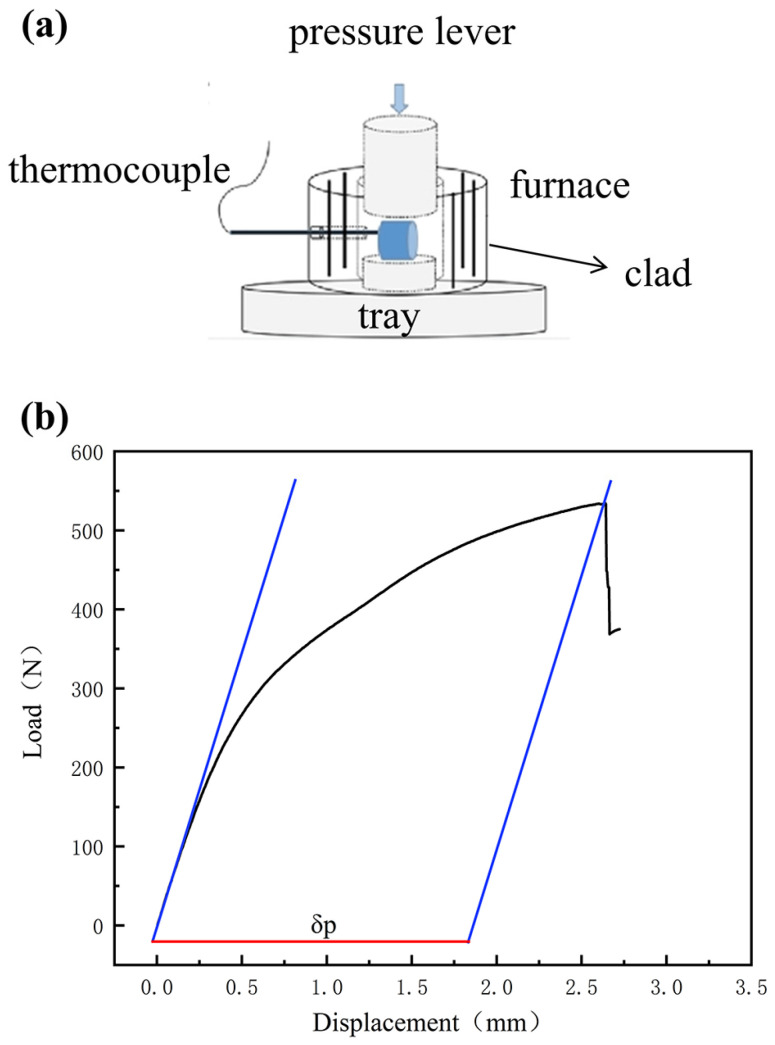
Schematic diagram of ring pressure test and offset strain calculation. (**a**) Schematic diagram of ring pressure test; (**b**) schematic diagram of offset strain calculation.

The accident tolerance of zirconium alloys is mainly reflected in their ability to maintain their original geometry under LOCA conditions; this is directly manifested by the retained ductility of zirconium alloys, referred to as residual ductility [17] and characterized by the offset strain in ring compression tests. To obtain the offset strain of zirconium alloys, the ring compression test is a widely accepted method. The specific test method was as follows: the quenched samples were cut into three 8 mm short samples, and the ring compression test was performed using a universal testing machine (Figure 2a shows a schematic diagram of the ring compression test principle). The test temperature was a constant temperature of 135 °C; the compression rate was 2 mm/min.

### 2.4. Sample Characterization

Hydrogen content determination: The hydrogen content in the samples was measured by the inert gas pulse infrared absorption method and neutron imaging.

Microstructural analysis: Before analysis of the samples after the test, sample preparation was required. The samples were embedded in epoxy resin to prepare cross-sectional samples of zirconium alloys. After grinding and polishing with 600#, 800#, and 1200# sandpapers, 9 μm diamond suspension, 3 μm diamond suspension, and alumina suspension in sequence, the samples were etched with a mixed etchant of concentrated nitric acid: hydrofluoric acid: water (volume ratio 9:2:9). Metallographic analysis of the cross-section of the test samples was performed using an optical microscope (model: Olympus CKX41); the microstructural morphology and element distribution of the zirconium alloys after the test were analyzed using a scanning electron microscope (SEM, model: JSM-7001F field-emission scanning electron microscope); the radial oxygen distribution of the zirconium alloy cross-section was determined and analyzed using electron probe microanalysis (EPMA, model: JXA-8230); samples were cut at the interface of the oxide film, coating, and matrix using a focused ion beam (FIB), and then the phase and element distribution of the samples were analyzed using a transmission electron microscope (TEM).

## 3. Results and Analysis

### 3.1. High-Temperature Steam Oxidation Kinetics Curves and Residual Ductility of Two Types of Claddings

**Figure 3:**
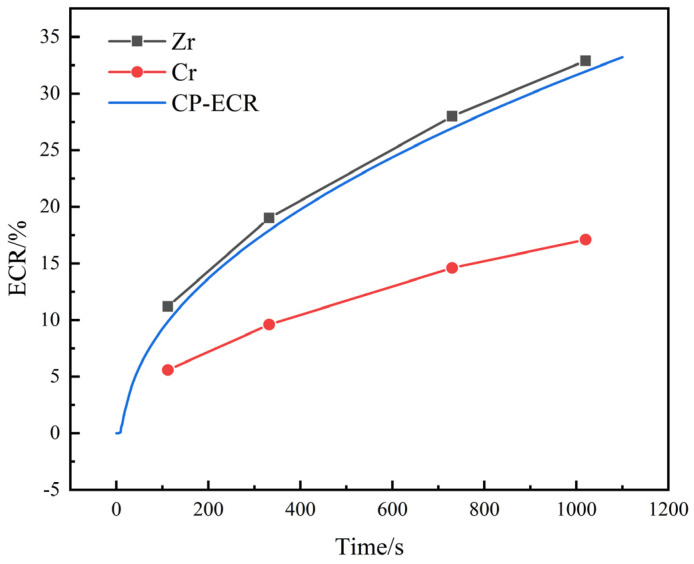
Kinetics of high-temperature steam oxidation of two kinds of cladding.

In Figure 3, the high-temperature steam oxidation kinetics curves of the two types of claddings are presented. It can be seen that with an increase in the oxidation duration, the oxidation degree of both types of claddings increases. The oxidation degree growth trend of uncoated claddings is basically consistent with the CP model formula for the high-temperature steam oxidation kinetics of zirconium alloys. The oxidation degree of Cr-coated claddings is much lower than that of uncoated claddings, approximately half that of the uncoated zirconium alloy claddings. This test was a double-sided oxidation experiment, and the inner surface of the Cr-coated claddings had no coating. Therefore, the weight gain of the Cr-coated claddings should be caused by oxidation of the inner surface, while the outer surface of the coating has almost no oxidation. The test results show that a Cr coating effectively prevents oxidation thinning of zirconium alloys.

**Figure 4:**
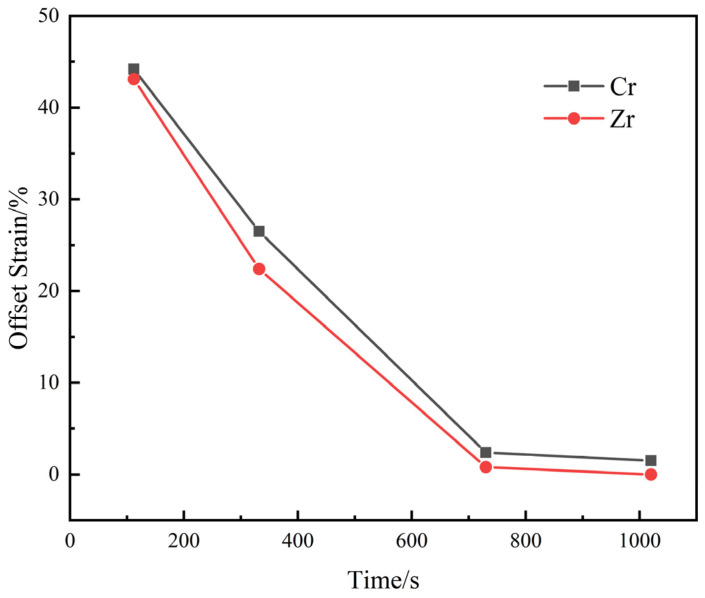
Comparison of residual plasticity of two types of cladding after LOCA.

Subsequently, the residual ductility was measured by the ring compression test, and the test results are shown in Figure 4. It can be found that the residual ductility of Cr-coated samples is only slightly higher than that of uncoated zirconium alloy claddings, which is inconsistent with their oxidation degree. According to the difference in oxidation degree between the two types of claddings, the residual ductility of Cr-coated claddings should be much higher than that of uncoated zirconium alloy claddings. However, this is not the case in reality, and it is speculated that it may be related to the absorption of oxygen or hydrogen during the test.

### 3.2. Oxygen Content and Distribution and Hydrogen Content and Distribution of Two Types of Claddings After Tests

**Figure 5:**
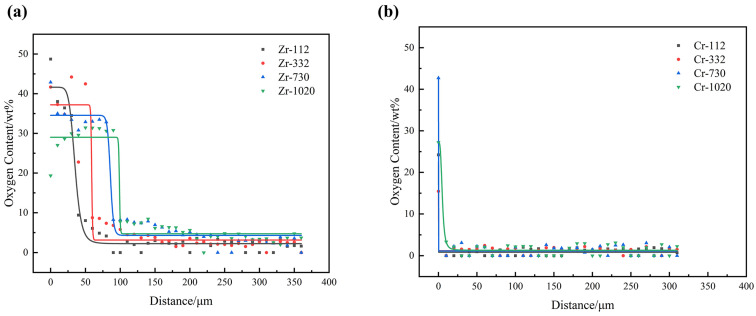
Oxygen content distribution: (**a**) zirconium alloy; (**b**) Cr-coated zirconium alloy.

Figure 5 shows the oxygen element distributions inside samples of the two types of zirconium alloy claddings with different oxidation degrees. With an increase in the oxidation degree, the internal oxygen content of uncoated claddings increases significantly, while the oxygen content of Cr-coated claddings almost does not change and is much lower than that of uncoated zirconium alloy claddings. The test results show that the presence of the Cr coating can effectively prevent oxidation of the zirconium alloy matrix and the diffusion of oxygen into the zirconium alloy matrix, and its abnormal residual ductility is unrelated to oxygen absorption.

**Figure 6:**
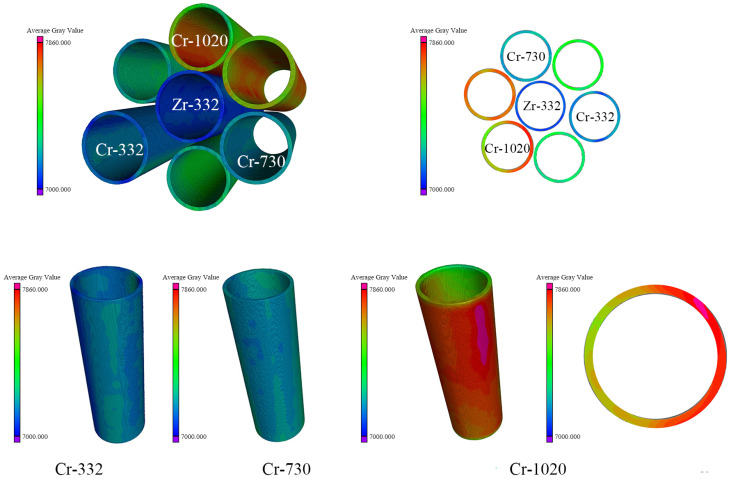
Neutron imaging results of hydrogen distributions in two types of cladding samples with several typical oxidation degrees.

Neutron imaging was used to characterize the overall distributions of hydrogen in the two types of claddings with several oxidation degrees after the simulated test, as shown in Figure 6. In the figure, blue represents a low hydrogen content, green represents medium, and red represents a high hydrogen content. It can be seen that the uncoated cladding samples are overall blue after the test, with low hydrogen content. The Cr-coated sample with an oxidation duration of 332 s shows a small part of green on the outer surface and blue in most areas, with a smaller green area on the inner surface, showing an overall blue color and slight hydrogen absorption. The Cr-coated sample with an oxidation duration of 730 s shows a large area of green on both the inner and outer surfaces, indicating more severe hydrogen absorption. Meanwhile, most areas of the sample with an oxidation duration of 1020 s are red and a small part is green, with the most severe hydrogen absorption. By observing the cross-sectional color of the Cr-coated sample with an oxidation duration of 1020 s, it can be found that some areas on the outer surface show a color corresponding to higher hydrogen content, while the inner surface shows a color corresponding to slightly lower hydrogen content; this indicates that hydrogen diffuses from the outer Cr coating area to the inner surface in the radial direction, while the axial diffusion of hydrogen does not show a regular pattern.

**Figure 7:**
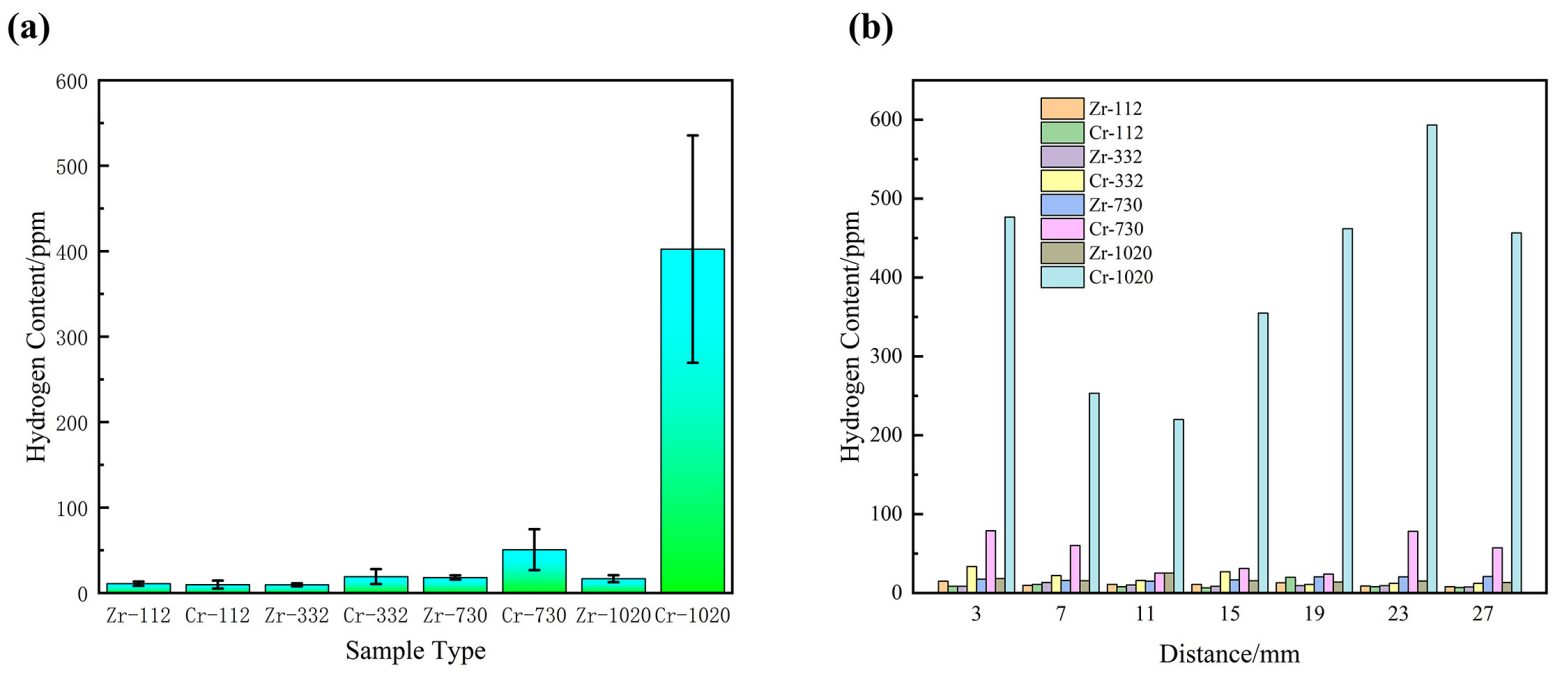
Hydrogen content and hydrogen distribution in two types of cladding samples with different oxidation degrees: (**a**) average hydrogen content; (**b**) axial hydrogen distribution.

Subsequently, the two types of samples were cut into seven 3 mm long segments corresponding to positions of 3 mm, 7 mm, 11 mm, 15 mm, 19 mm, 23 mm and 27 mm on the original samples. The hydrogen content of each segment was tested to characterize the hydrogen content and distribution of the two claddings under four oxidation durations. Figure 7a shows the average hydrogen contents of the seven measured positions, and Figure 7b lists the hydrogen content value at each single position. This measurement technique is a destructive testing method. It can be found that when the oxidation duration is 112 s, the hydrogen content of the two types of claddings is basically the same, with almost no increase in hydrogen content. As the oxidation duration increases, the hydrogen content of the Cr-coated claddings rises sharply, with an average hydrogen concentration of 402 ppm at an oxidation duration of 1020 s; the hydrogen content of uncoated claddings almost does not change, fluctuating between 10 and 20 ppm, which is the same as the initial hydrogen content of zirconium alloy (<20 ppm). By analyzing the hydrogen distribution of the two types of claddings, it can be seen that when the oxidation duration is 112 s, the hydrogen content of both types of claddings is low and uniformly distributed. As the oxidation duration increases, the hydrogen content of Cr-coated claddings increases significantly and is extremely unevenly distributed. When the oxidation duration is 1020 s, the local peak hydrogen content can reach 593 ppm, while the hydrogen content of uncoated zirconium alloys is uniformly distributed, fluctuating between 10 and 25 ppm. The test results show that severe hydrogen absorption occurs in Cr-coated claddings during simulated LOCA tests, resulting in abnormal residual ductility. The Cr-coated claddings are analyzed through relevant microstructural analysis results in the following sections.

### 3.3. SEM Analysis of Zirconium Alloy Microstructure

**Figure 8:**
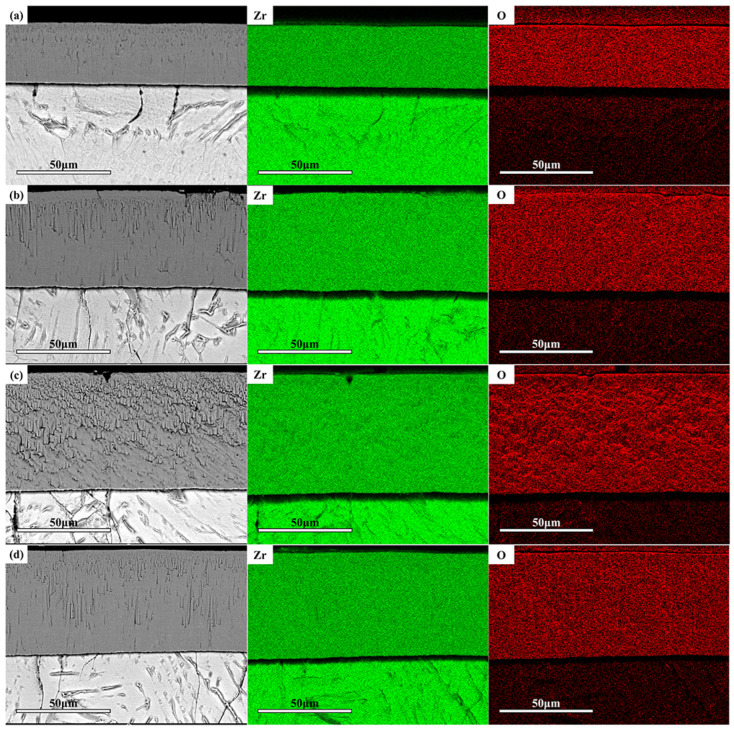
SEM analysis of uncoated cladding samples with different oxidation degrees: (**a**) Zr-112; (**b**) Zr-332; (**c**) Zr-730; (**d**) Zr-1020.

Figure 8 shows the cross-sectional microstructure and element distribution of uncoated zirconium alloy claddings after the test. It can be seen that the cross-sectional structures of samples with different oxidation degrees are basically the same except for the thickness of the oxide film, showing a typical “sandwich-like” microstructure after LOCA tests of zirconium alloys. This microstructure consists of an outermost dense ZrO_2_ oxide film, a middle O-enriched α-Zr(O) layer, and an innermost quenched prior-β-Zr layer. The ZrO_2_ oxide film shows a uniform and dense morphology, and the spallation pits on its surface are caused by grinding and polishing during sample preparation, with no pores or cracks in the oxide film.

**Figure 9:**
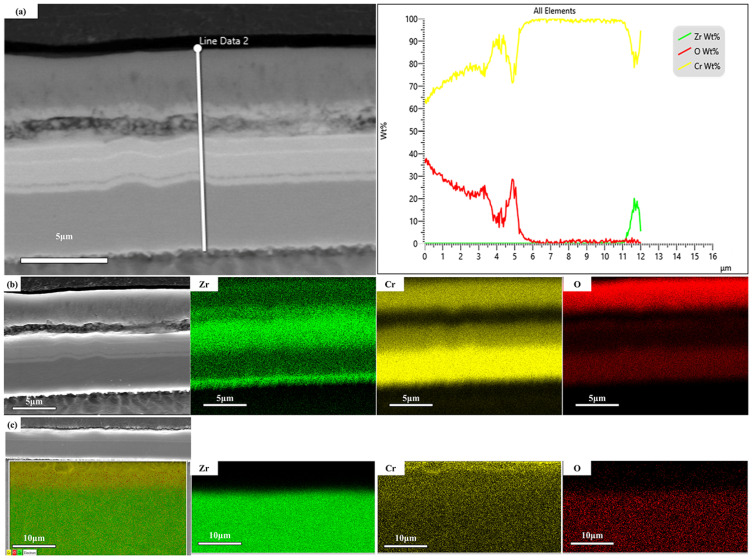
The cross-sectional microstructure and elemental distribution of Cr-112 after the test: (**a**) EDS line scan; (**b**) EDS maps of the coating; (**c**) EDS maps of the substrate.

Figure 9 shows the cross-sectional microstructure and element distribution of Cr-coated zirconium alloy cladding specimens after testing at an oxidation duration of 112 s. It can be seen that the Cr coating undergoes little oxidation, with only partial oxidation on the surface. The intermediate Zr element layer is produced by diffusion of Zr elements along the grain boundaries of the Cr coating to the surface and reduction of the chromium oxide layer during high-temperature oxidation, which has been reported in many studies [19,20,21]. Subsequent to this is the Cr coating still connected to the zirconium alloy matrix, in which a certain amount of oxygen diffusion exists. Finally, the zirconium alloy matrix part shows a certain interdiffusion of Cr-Zr elements in the part in contact with the Cr coating, which has also been mentioned in many studies [19,20,21].

**Figure 10:**
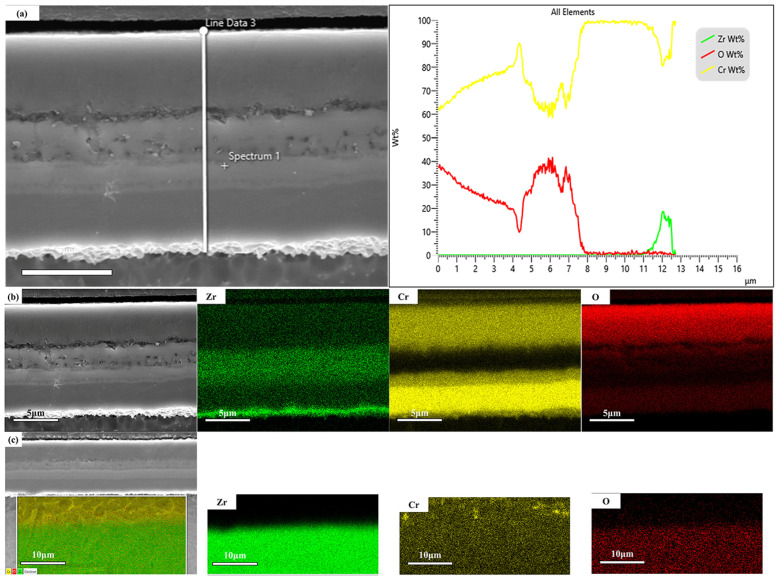
The cross-sectional microstructure and elemental distribution of Cr-332 after the test: (**a**) EDS line scan; (**b**) EDS maps of the coating; (**c**) EDS maps of the substrate.

Figure 10 shows that when the oxidation duration is 332 s, oxidation of the Cr coating increases, and more pores appear in the subsequent Zr element layer. More oxygen elements appear in the upper layer, and a Cr-Zr element mixed layer appears in the lower layer. Compared with that for an oxidation duration of 112 s, the lower Cr element layer also has a higher oxygen content. Finally, the zirconium alloy matrix part shows more interdiffusion of Cr-Zr elements in the part in contact with the Cr coating, and more Cr element agglomeration also exists in positions far from the Cr-Zr contact area.

**Figure 11:**
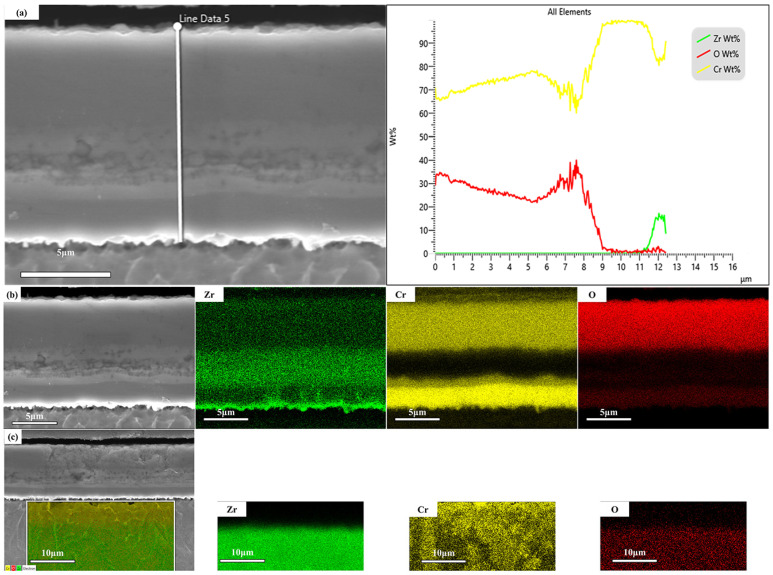
The cross-sectional microstructure and elemental distribution of Cr-730 after the test: (**a**) EDS line scan; (**b**) EDS maps of the coating; (**c**) EDS maps of the substrate.

Figure 11 shows that when the oxidation degree is an oxidation duration of 730 s, the oxidation of the Cr coating is more obvious, but it is not completely oxidized. More pores and cracks appear in the subsequent Zr element layer. Similarly, more oxygen elements appear in the upper layer, and a Cr-Zr element mixed layer appears in the lower layer. Compared with that for an oxidation duration of 332 s, the lowest Cr element layer is thinner and has a higher oxygen content. Finally, the zirconium alloy matrix part shows a great deal of interdiffusion of Cr-Zr elements in the part in contact with the Cr coating; a large amount of Cr element is also distributed in positions far from the Cr-Zr contact area, with severe Cr element agglomeration in some positions.

**Figure 12:**
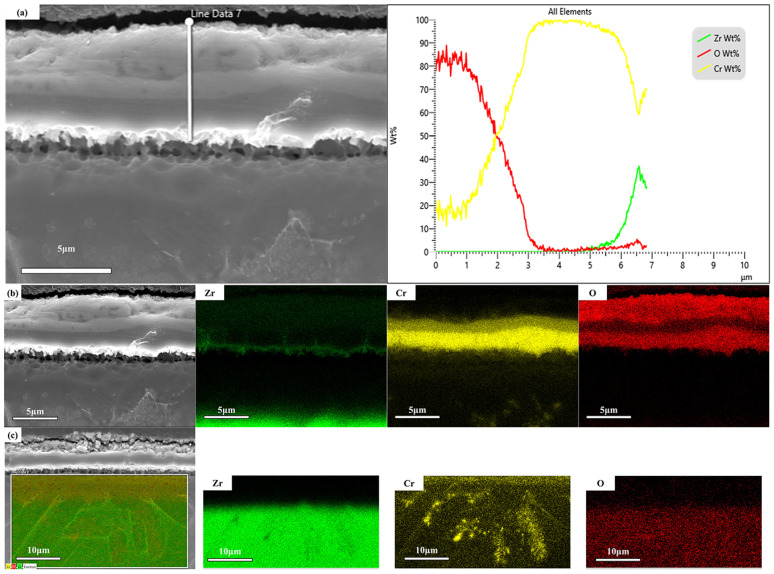
The cross-sectional microstructure and elemental distribution of Cr-1020 after the test: (**a**) EDS line scan; (**b**) EDS maps of the coating; (**c**) EDS maps of the substrate.

Figure 12 shows that when the oxidation degree is an oxidation duration of 1020 s, the Cr coating undergoes extremely severe oxidation, and the chromium oxide layer is thin. This is caused by the volatilization of chromium oxide at a high temperature of 1200 °C. The subsequent Zr element layer is also very thin, almost invisible in the figure. Compared with that for an oxidation duration of 730 s, the lowest Cr element layer has a very high oxygen content, basically the same as that in the chromium oxide layer. Finally, the zirconium alloy matrix part shows an extremely large amount of interdiffusion of Cr-Zr elements in the part in contact with the Cr coating, and very severe Cr element agglomeration along the grain boundaries also exists in positions far from the Cr-Zr contact area.

From the above test results, it can be seen that the overall cross-sectional structure of samples with different oxidation degrees is basically the same, consisting of an outermost chromium oxide layer, a subsequent Zr element layer, a Cr element layer, and the internal zirconium alloy matrix. However, the specific thickness, morphology, and element distribution of each layer are different.

### 3.4. TEM Analysis of Zirconium Alloy Microstructure

**Figure 13:**
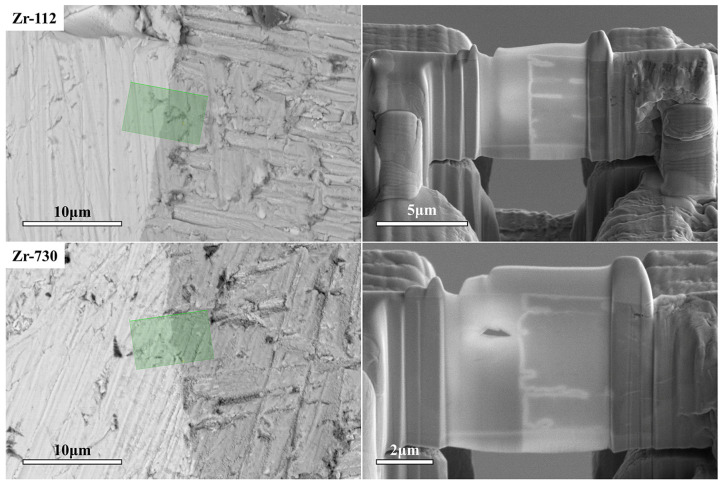
Uncoated zirconium cladding FIB sampling.

Figure 13 shows the FIB sampling that was performed at the interface between the oxide film and the zirconium alloy matrix of uncoated zirconium alloy claddings with oxidation durations of 112 s and 730 s after the test, followed by TEM analysis of these samples.

**Figure 14:**
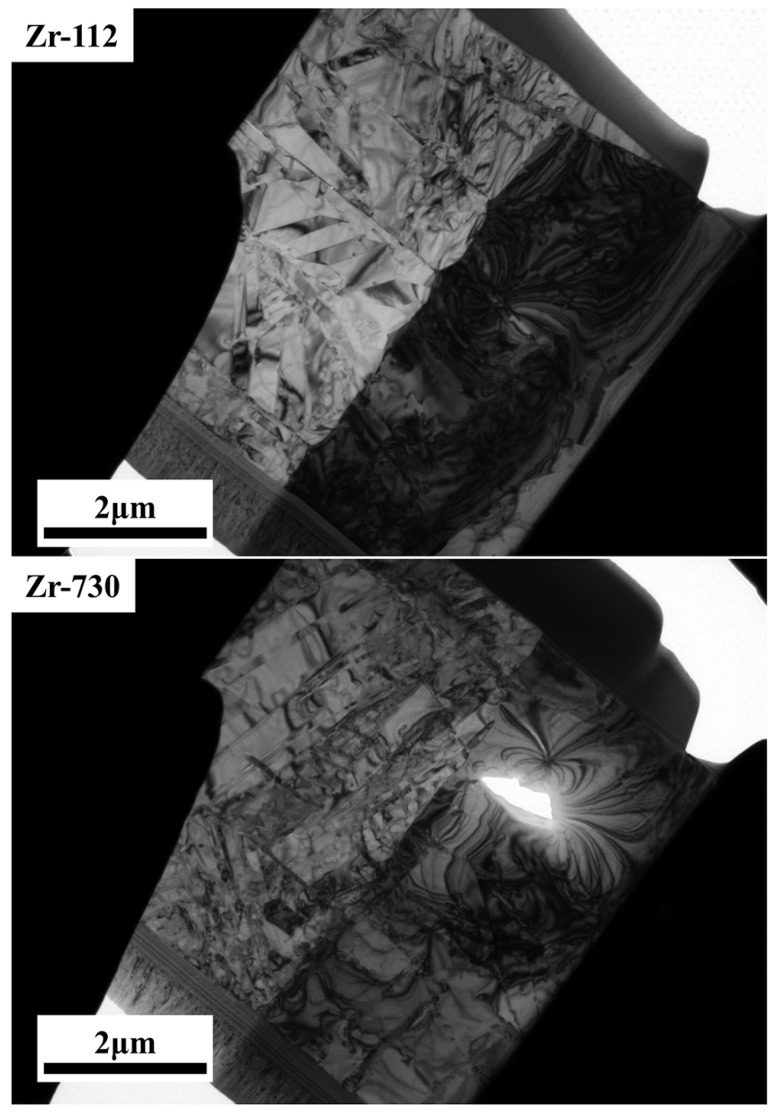
Morphology of uncoated zirconium alloy cladding oxide film and matrix.

Figure 14 shows the analysis of the morphology at the interface between the oxide film and the matrix. It can be seen that the sample has no other layered structures except for the oxide film and the matrix. The interface between the oxide film and the matrix is closely connected, the oxide film grains are small, the morphology is dense, and there are no pores.

**Figure 15:**
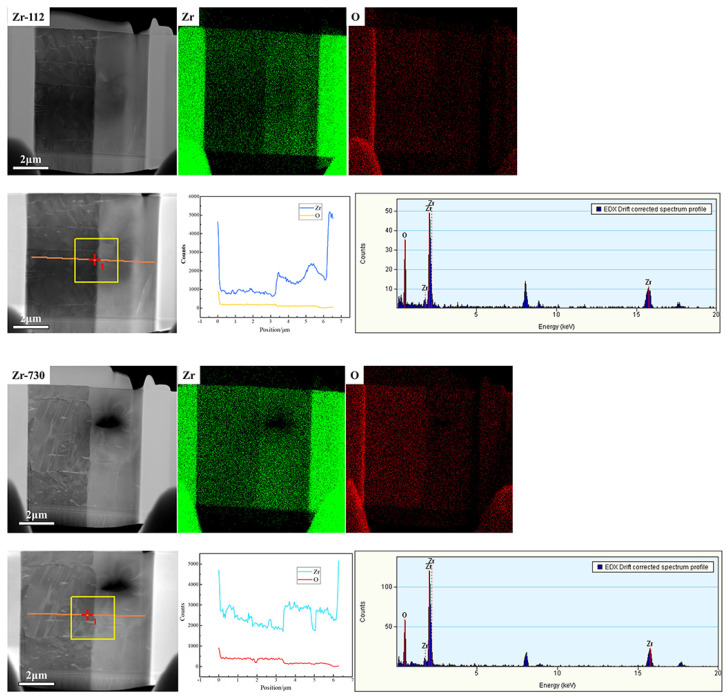
Distribution of elements at join of uncoated zirconium alloy cladding oxide film and matrix.

The analysis of the element composition distributions of the samples is shown in Figure 15. It can be seen that only Zr and O elements exist in the samples, and no other elements are present. The oxygen content in the oxide film is high, and a certain amount of oxygen also exists in the zirconium alloy matrix. However, the boundary between the oxide film and the zirconium alloy matrix is clear, and there is no intrusion of the oxide film.

**Figure 16:**
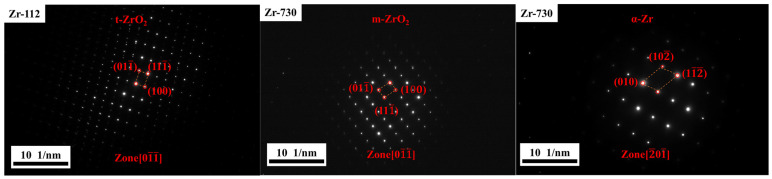
SAED diagram of uncoated zirconium alloy cladding oxide film and substrate.

The phases of the oxide film and the matrix were determined by SAED in the oxide film and the zirconium alloy matrix, as shown in Figure 16. It was found that Zr-112 is tetragonal ZrO_2_ and Zr-730 is monoclinic ZrO_2_, which is consistent with the monoclinic and tetragonal phases of zirconium alloy oxide films after LOCA tests reported in the literature. The matrix is hexagonal close-packed α-Zr.

**Figure 17:**
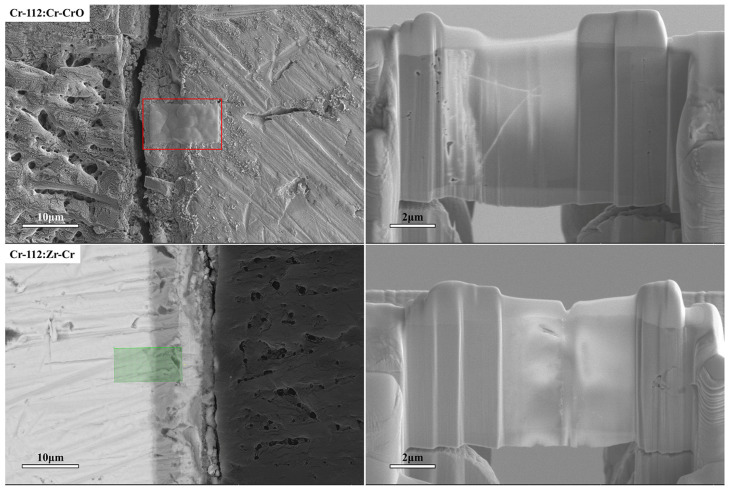
FIB sampling of Cr-coated zirconium alloy cladding.

Figure 17 shows the FIB sampling that was performed at the interface between the oxide film and the unoxidized Cr coating and that between the unoxidized Cr coating and the zirconium alloy matrix in the cross-sectional structure of Cr-coated zirconium alloy claddings after the simulated test, followed by TEM analysis of these samples.

**Figure 18:**
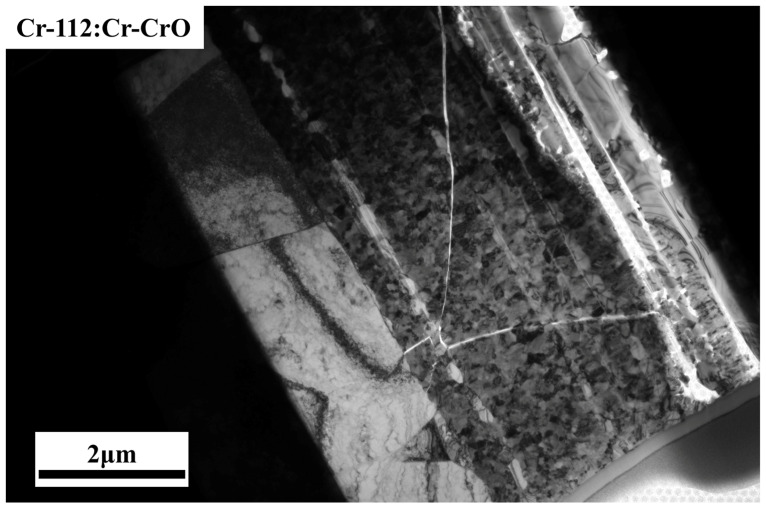
Morphology of Cr-CrO junction of Cr-coated zirconium alloy cladding.

Figure 18 shows the analysis of the morphology at the interface between the oxide film and the unoxidized Cr coating. It can be seen that the sample presents a three-layer structure morphology, corresponding to the SEM results. The outermost layer has the coarsest grains, the middle layer is a fine-grain structure, and the innermost layer fractured during sample preparation but also shows a relatively coarse grain structure.

**Figure 19:**
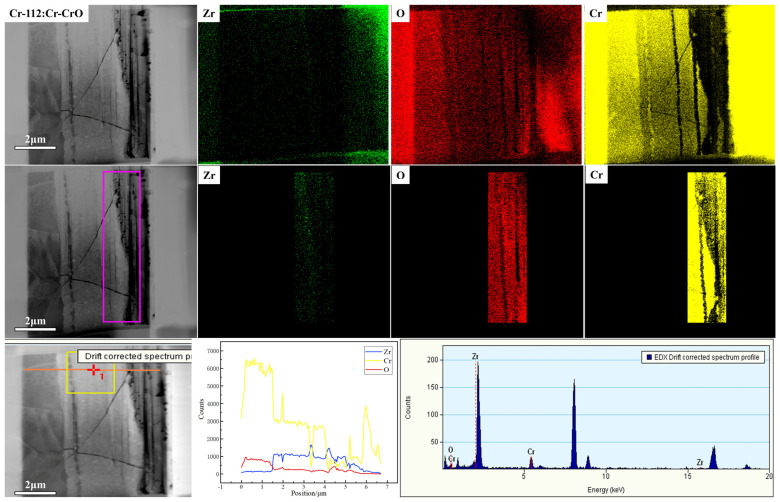
Element distribution at Cr-CrO junction of coated zirconium alloy cladding.

Figure 19 shows the analysis of the element composition distribution at the interface between the oxide film and the unoxidized Cr coating. It can be seen that the outermost layer is a chromium oxide layer, and the middle layer and the innermost layer are mixed layers of Cr, Zr, and O elements. The middle layer contains a little more Zr elements, and the innermost layer contains more Zr elements.

**Figure 20:**
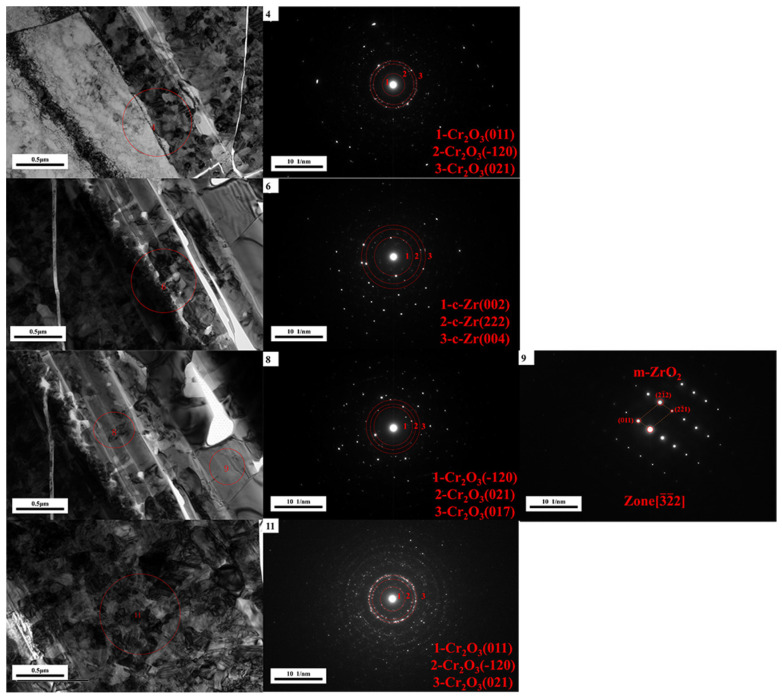
SAED diagram of chromium-coated zirconium cladding oxide film and chromium-coated substrate.

The phases of the three layers in the sample were determined by SAED, as shown in Figure 20. It can be seen that SAED pattern 4 shows that the main substance in the outermost layer is Cr_2_O_3_; SAED patterns 6 and 11 show that the substances existing in the middle layer are Cr_2_O_3_ and Zr; and SAED patterns 8 and 9 show that the substances existing in the innermost layer are also Cr_2_O_3_ and ZrO_2_. The test results show that after the test, the microstructure of the Cr coating is relatively complex, with various substances mixed in each layer; this may be one of the reasons for hydrogen accumulation.

**Figure 21:**
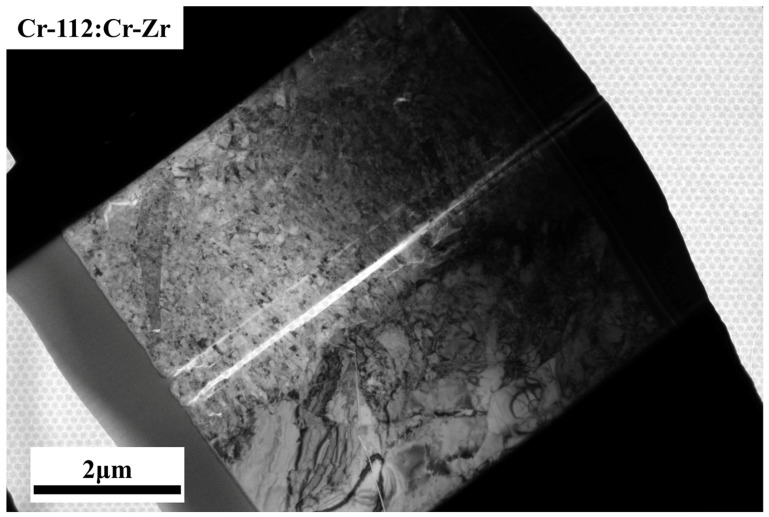
Morphology of Cr-Zr binding of chromium-coated zirconium alloy cladding.

**Figure 22:**
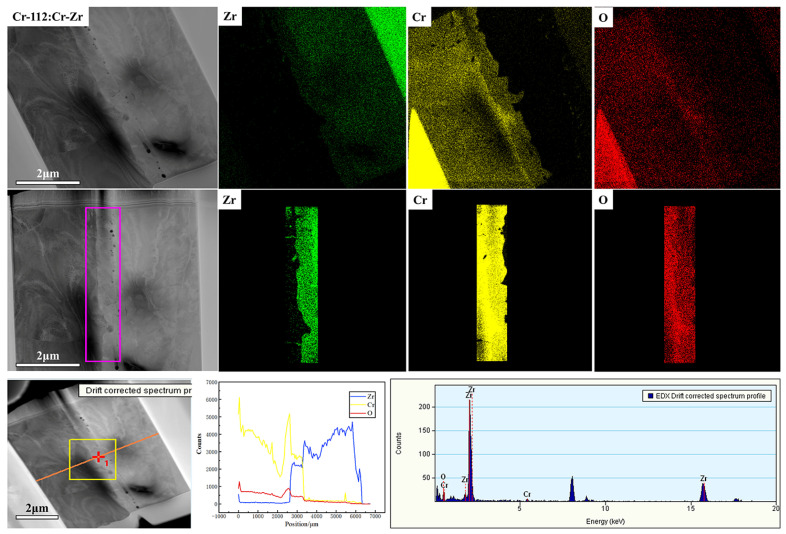
Element distribution at Cr-Zr junction of chrome-coated zirconium alloy cladding.

**Figure 23:**
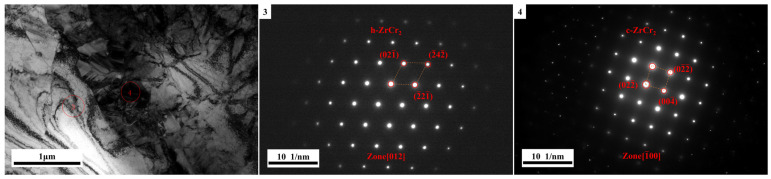
SAED diagram at Cr-Zr junction of chrome-coated zirconium alloy cladding.

Figure 21 shows the analysis of the morphology at the interface between the unoxidized Cr coating and the zirconium alloy matrix. It can be seen that the sample presents a two-layer structure morphology, with the unoxidized Cr coating on one side and the zirconium alloy matrix on the other side. The Cr element layer has small grains and is relatively dense, while the Zr element layer has large grains. The analysis of the element composition distribution of the sample is shown in Figure 22. It can be seen that a small amount of oxygen also exists in the Cr coating, the boundary between the Cr and Zr layers is irregular, and there is an obvious interdiffusion phenomenon between Cr and Zr elements. The phase of the substance at the boundary in the sample was determined by SAED, as shown in Figure 23. It can be seen that the boundary is ZrCr_2_ intermetallic compound.

### 3.5. Analysis and Discussion

**Figure 24:**
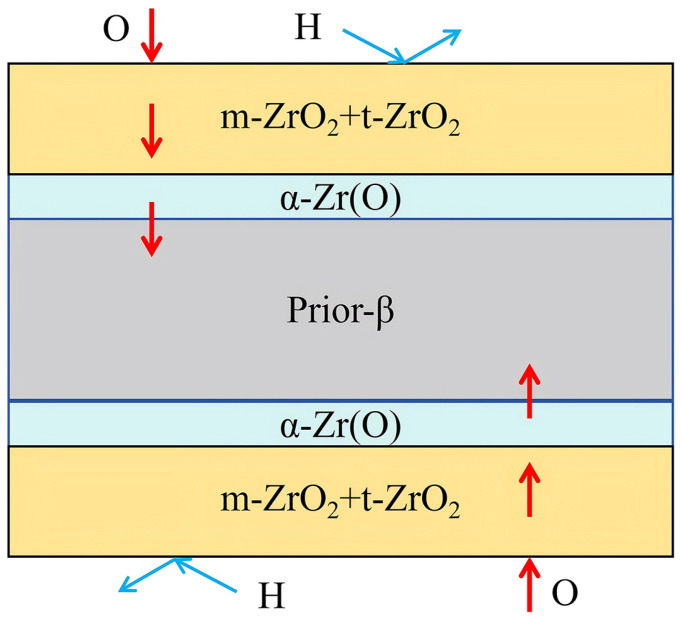
Schematic diagram of structure and internal elements of uncoated cladding in LOCA.

Figure 24 shows a schematic diagram of the microstructure and internal element change law of uncoated claddings during simulated LOCA tests. The change in their microstructure and elements is relatively simple. It can be determined that the sample is divided into three layers during the test: a ZrO_2_ layer, an α-Zr(O) layer, and a quenched prior-β phase layer. The oxide film contains monoclinic and tetragonal phases, and its oxygen barrier performance is poor. However, no severe hydrogen accumulation occurs during the test, which is consistent with the results reported in a large number of studies on LOCA research of zirconium alloys. In the literature, the hydrogen absorption rate is generally expressed by the formula $f_{H}=\frac{H_{absorbed}}{H_{generated}}$, and the ZrO_2_ layer’s low hydrogen absorption rate is related to its good hydrogen barrier performance. The oxygen vacancy structure in ZrO_2_ constitutes hydrogen traps, which can effectively capture hydrogen and prevent its diffusion into the zirconium alloy matrix [22].

**Figure 25:**
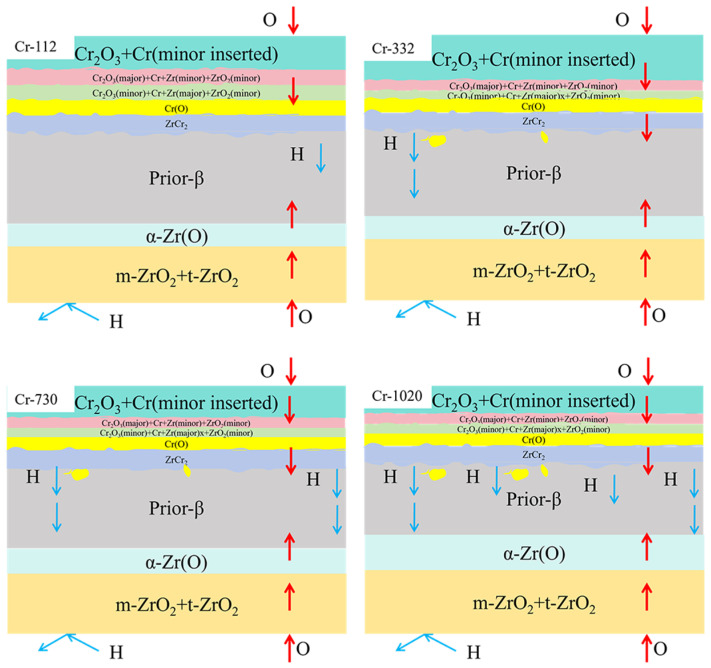
Schematic diagram of structure and internal elements of CR-coated claddings with different oxidation degrees in simulated LOCA tests.

Figure 25 shows a schematic diagram of the microstructure and internal element change law of Cr-coated claddings with different oxidation durations during simulated LOCA tests. The change in their microstructure and elements is very complex. From the SEM analysis results, when the oxidation duration is 112 s, the coating is divided into multiple layers. The TEM analysis results also show that the coating is divided into a chromium oxide + inserted Cr layer, a layer with a large amount of chromium oxide + Cr + a small amount of Zr and ZrO_2_, a layer with a large amount of Zr + a small amount of chromium oxide + Cr and ZrO_2_, an oxygen-containing Cr layer, and a ZrCr_2_ intermetallic compound layer. From the EPMA results, the Cr-coated claddings have excellent oxygen barrier performance. At the same time, when the oxidation duration is 112 s, no severe hydrogen accumulation occurs in the Cr-coated zirconium alloy during the test, which may be related to the low oxidation degree and less hydrogen produced by oxidation.

When the oxidation duration is 332 s, the basic structure is the same as that when the oxidation duration is 112 s, but the oxide layer is thicker, the oxygen-rich Cr layer is thinner, and Cr element agglomeration exists in the quenched prior-β layer. A certain degree of hydrogen accumulation occurs during the test. Based on the research results on the microstructure, the reasons for hydrogen accumulation can be analyzed. It can be found that there are small amounts of Cr inserted phases in the oxide film of Cr-coated claddings. Although chromium oxide has excellent hydrogen barrier performance, the existence of Cr inserted phases provides channels for hydrogen to diffuse into the material matrix. In other layers, the composition is more complex, and there are metallic phases. Hydrogen continuously diffuses into the matrix along the grain boundaries of the metallic phases, resulting in hydrogen accumulation in the Cr coating.

When the oxidation duration is 730 s, its basic structure still does not change. However, it can be seen that there are a large number of cracks in the middle layer (a layer with a large amount of chromium oxide + Cr + a small amount of Zr and ZrO_2_) and the layer with a large amount of Zr + a small amount of chromium oxide + Cr and ZrO_2_, which further facilitates the accumulation of hydrogen in the matrix. At the same time, a longer oxidation duration also produces more hydrogen, resulting in more hydrogen accumulation in the cladding matrix.

When the oxidation duration is 1020 s, it can be seen that the oxide layer becomes thinner instead, which may be caused by the volatilization of chromium oxide at a high temperature of 1200 °C [23]. At the same time, the intermediate Zr-containing layer becomes thinner. The thinner oxide layer further reduces the resistance to hydrogen accumulation in the cladding matrix. At the same time, a longer oxidation duration also produces more hydrogen, resulting in an extremely large amount of hydrogen accumulation in the Cr-coated cladding matrix when the oxidation duration is 1020 s.

## 4. Conclusions

In this study, simulated LOCA tests with different oxidation durations were performed on uncoated and Cr-coated claddings. The internal elements of the claddings were analyzed by EPMA, neutron imaging, and the inert gas pulse infrared absorption method. The evolution of the microstructure of the samples was analyzed by SEM and TEM. The hydrogen behavior and mechanism of Cr-coated zirconium alloy claddings during simulated LOCA tests were explored, and the following conclusions were drawn:

The test results showed that uncoated claddings form a relatively simple microstructure during simulated LOCA tests. Dense zirconia formed on the outer surface acts as a good hydrogen barrier material, preventing the diffusion of hydrogen into the zirconium alloy matrix. Cr-coated claddings form a complex microstructure during simulated LOCA tests: the outermost layer is a chromium oxide + inserted Cr layer, the second layer has a large amount of chromium oxide + Cr + a small amount of Zr and ZrO_2_, the third layer has a large amount of Zr + a small amount of chromium oxide + Cr and ZrO_2_, the fourth layer is an oxygen-containing Cr layer and ZrCr_2_ intermetallic compound layer, and the innermost layer is the quenched prior-β phase. As the smallest atom, hydrogen easily diffuses through the interstitial sites of metal atoms and can thus penetrate metals. In contrast, for oxides, hydrogen barely permeates intact oxide crystals due to the hindrance effect of O-H bonds on hydrogen diffusion. This is the primary reason why oxides are commonly employed as hydrogen permeation barrier coatings [24,25]. To penetrate oxides, hydrogen must mainly travel through cracks and pores. In this study, distinct cracks, pores, and metallic phases were observed in parts of the chromium oxide layer. Therefore, it is inferred that these features serve as the main pathways for hydrogen permeation, and such microstructures may be a potential factor contributing to hydrogen enrichment in Cr-coated claddings under LOCA conditions. With extension of the oxidation duration, the initiation and propagation of cracks within the coating, volatilization of chromium oxides, and increased hydrogen generation from prolonged oxidation are all likely to be key factors responsible for the elevated hydrogen content in Cr coatings.

## Figures and Tables

**Table 1:** Chemical composition of Zr-Sn-Nb alloy (wt.%).

Sn	Nb	Fe	O	Cr	Ni	Zr
1.1	1.1	0.1	0.12	<0.01	<0.01	Bal.

## Data Availability

The original contributions presented in this study are included in the article. Further inquiries can be directed to the corresponding author.
